# Antibacterial, injectable, and adhesive hydrogel promotes skin healing

**DOI:** 10.3389/fbioe.2023.1180073

**Published:** 2023-06-02

**Authors:** Zilong Fang, Tao Lin, Shuai Fan, Xing Qiu, Ziqing Zhong, Ganghua Yang, Jianqiu Yang, Guoqing Zhang, Yang Feng, Fanrong Ai, Qingming Shi, Wenbing Wan

**Affiliations:** ^1^ The Second Clinical Medical School, Nanchang University, Nanchang, Jiangxi, China; ^2^ Oujiang Laboratory (Zhejiang Lab for Regenerative Medicine, Vision & Brain Health), Wenzhou, Zhejiang, China; ^3^ Department of Orthopedic Surgery, Affiliated Zhongshan Hospital of Dalian University, Dalian, Liaoning, China; ^4^ School of Advanced Manufacturing, Nanchang University, Nanchang, Jiangxi, China; ^5^ Department of Orthopedic Surgery, The Second Affiliated Hospital of Nanchang University, Nanchang, Jiangxi, China

**Keywords:** antibacterial, injectable, promoting healing, hydrogel, wound dressing

## Abstract

With the development of material science, hydrogels with antibacterial and wound healing properties are becoming common. However, injectable hydrogels with simple synthetic methods, low cost, inherent antibacterial properties, and inherent promoting fibroblast growth are rare. In this paper, a novel injectable hydrogel wound dressing based on carboxymethyl chitosan (CMCS) and polyethylenimine (PEI) was discovered and constructed. Since CMCS is rich in -OH and -COOH and PEI is rich in -NH_2_, the two can interact through strong hydrogen bonds, and it is theoretically feasible to form a gel. By changing their ratio, a series of hydrogels can be obtained by stirring and mixing with 5 wt% CMCS aqueous solution and 5 wt% PEI aqueous solution at volume ratios of 7:3, 5:5, and 3:7. Characterized by morphology, swelling rate, adhesion, rheological properties, antibacterial properties, *in vitro* biocompatibility, and *in vivo* animal experiments, the hydrogel has good injectability, biocompatibility, antibacterial (*Staphylococcus aureus*: 56.7 × 10^7^ CFU/mL in the blank group and 2.5 × 10^7^ CFU/mL in the 5/5 CPH group; *Escherichia coli*: 66.0 × 10^7^ CFU/mL in the blank group and 8.5 × 10^7^ CFU/mL in the 5/5 CPH group), and certain adhesion (0.71 kPa in the 5/5 CPH group) properties which can promote wound healing (wound healing reached 98.02% within 14 days in the 5/5 CPH group) and repair of cells with broad application prospects.

## 1 Introduction

It is difficult to avoid skin trauma in everyone’s life, and serious skin trauma may even lead to death. Skin wound dressing is very important for wound repair and protection during wound healing (Liang et al., 2021). Establishing how to design a new wound dressing has been an urgent issue in modern medical field technology (Guo and Ma, 2018). Among the novel dressing materials developed in recent years, novel wound dressings including 3D-printed scaffolds, sponges, and hydrogels have been developed. The dressing materials developed in recent years include 3D-printing scaffolds, sponges, and hydrogels (Xu et al., 2018; Cui et al., 2020; Biranje et al., 2022). Among these materials, hydrogels can not only form a good physical barrier in the wound but also provide a humid environment to promote wound healing (Gong et al., 2013; Fan et al., 2014). In addition, research on the electrospinning fiber patch and microneedle array patch have also made important contributions to the development of wound dressings (Iacob et al., 2020; Hu et al., 2023). An antibacterial effect of traditional hydrogel dressing is mainly due to antibiotics or other antibacterial substances in the hydrogel matrix (Kumar et al., 2012; Song et al., 2021; Huang et al., 2022; Zhao et al., 2022). Adding other antibacterial substances (such as antibiotics or antimicrobial peptides) not only increases the synthesis method and cost of hydrogels but also may lead to inconsistent release of antibacterial substances, showing an inconsistent antibacterial activity (Cao et al., 2021a). Hydrogels with inherent antibacterial properties are more attractive because of their consistent antibacterial activity (Yadollahi et al., 2015; Qu et al., 2018a). Another challenge is that, in new dressing materials, the addition of growth factors to promote wound healing makes the synthesis of wound dressing complex and expensive (Qian et al., 2020; Jian et al., 2022). Hydrogels with inherent ability to promote wound cell proliferation also become very attractive.

Hydrogels based on chitosan are widely used in tissue engineering due to good biocompatibility, biodegradability, and inherent ability to promote the growth of fibroblasts (Nada et al., 2019; Fu et al., 2022; Guo et al., 2022). However, low solubility and reactivity of chitosan limit its role in hydrogel materials. As a derivative of chitosan, carboxymethyl chitosan (CMCS) can effectively improve the physical and chemical properties of chitosan. It has good solubility and reactivity and can retain good biocompatibility and promote healing (Yin et al., 2018; Cao et al., 2021b). It has been previously found that the copolymer formed by chemically modifying polyethylenimine (PEI) with CMCS has a strong ability to complex with DNA and can form nanoparticles, which can be used as an effective and safe non-viral vector (Liu et al., 2016). A CMCS- and PEI-blended membrane was used for the separation of a CO_2_/N_2_ mixture through physical interaction (Shen et al., 2013). Liming Bian’s team reported a polyethylenimine/polyacrylic acid (PEI/PAA) hydrogel, which was formed through strong hydrogen-bond interactions between PEI and PAA (Peng et al., 2021). PEI has strong cell adhesion, and because of its rich amino group, it has good antibacterial properties through a cationic bactericidal mechanism (Yang et al., 2022). Therefore, this study attempted to combine CMCS and PEI to form a hydrogel, thereby exerting their respective roles.

In conclusion, this paper used the strong physical interaction between CMCS and PEI to form a new CMCS/PEI (CPH) hydrogel and to explore the different ratios of CMCS/PEI (7:3; 5:5; and 3:7) and characterization of biocompatibility, adhesion, and antibacterial properties of chitosan as a biological dressing. In this study, chitosan is found to have a wide range of natural sources with low cost, which can easily be used to prepare hydrogel, and the hydrogel has good biocompatibility and antibacterial properties to promote cell growth with certain adhesion and injectability. Therefore, this CPH hydrogel has a good application prospect as wound dressing.

## 2 Materials and methods

### 2.1 Materials

Carboxymethyl chitosan (degree of substitution: ≥90%, isoelectric point: 3–4; McLean), polyethylenimine (molecular weight: 70,000; 50 wt%; McLean), DMEM, and PBS were purchased from Aladdin.

### 2.2 Preparation of CHP hydrogels

We mixed 5 wt% of CMCS (about 240,000 Mw) and 5 wt% of PEI (about 70,000 Mw) according to the following volume proportion (7:3; 5:5; and 3:7) to form the gel. After stirring the material at room temperature (25°C) in a magnetic stirrer at 800 rpm for 6 h, the mixture was immediately immersed in liquid nitrogen for about 20 min and freeze-dried to remove moisture. Finally, the dried solid was ground up using a mortar and pestle to obtain CMCS/PEI powder for preservation. We then took 0.5 g of hydrogel powder, added it to 5 mL of PBS at 37°C and stirred to dissolve for 1 h, and then placed the solution in a 37°C incubator and incubated for 24 h to wait for complete gelation.

### 2.3 SEM and FTIR characterization

A field-emission scanning electron microscope was used to observe the morphology of the CPH hydrogel (Zhao et al., 2018). Prior to observation, the hydrogel was freeze-dried and coated with gold. Hydrogel functional groups were determined by Fourier transform infrared (FTIR) spectroscopy (Nicolet 6,700) (Qu et al., 2018b). The freeze-dried hydrogels were triturated with liquid nitrogen and then pressed with potassium bromide (KBr) and analyzed in the spectral range of 4,000–400 cm^−1^.

### 2.4 Porosity, water absorption, and zeta potential of hydrogels

The liquid displacement method was used to measure the porosity of the hydrogels (Ponrasu et al., 2018). The initial weight m_1_ (g) and volume (mm^3^) of lyophilized hydrogel samples were tested. Subsequently, the samples were completely immersed in anhydrous ethanol solution until the mass remained constant at room temperature and the liquid gravimetric m_2_ (g) of the material surface was washed off with a filter paper. The porosity was calculated as follows: $Porosity\left(\%\right)=\frac{m2-m1}{\rho V}x100\%$ , where the parameter ρ represents the density of anhydrous ethanol. Each material was measured three times. The water absorption of the hydrogel was calculated according to the formula: $water\;absorption=\frac{W_{s}-W_{i}}{W_{i}}x100\%$ , where W_i_ (g) and W_s_ (g) are the initial weight of the hydrogel and the swollen weight at different times, respectively. All experiments were carried out three times, taking 2 mg of freeze-dried powder and diluting it in 3 mL of ultrapure water and then using a multi-angle particle size for analysis by means of a high-sensitivity zeta potential analyzer (model: NanoBrook Omni) for testing (Peng et al., 2021).

### 2.5 Hydrogel adhesion

The adhesion of the hydrogel was determined by means of shear tests (Liu et al., 2022). Fresh pigskin purchased from the farmers’ market was cut into a 3 cm long and 1 cm wide strip and soaked in 0.9% normal saline for 3–4 h for later use. The hydrogel was made into a disc with a diameter of 1 cm, and after the pigskins were wiped clean with water, the hydrogel was adhered between two pieces of pigskin, and its adhesion strength was measured by a universal testing machine [CMT6104, Metex Industrial Systipes (China) Co., LTD.]. All experiments were performed in triplicate.

### 2.6 Hydrogel rheological properties and injectability

A rheometer with a 40 mm flat plate and 2.0 mm gap was used for rheological testing (Li et al., 2017; Cao et al., 2021b). The rheology test required that the hydrogel sample should be completely gelled in an incubator at 37°C for 24 h in advance. The storage modulus (G′) and loss modulus (G^″^) of the hydrogel were determined by oscillating frequency scanning measurements at 10% strain, a shear frequency of 0.1–100 rad/s, and a temperature of 25°C. In injectability experiments of hydrogels, the hydrogel was dyed by directly mixing rhodamine B with the hydrogel; we added 0.1 mL of rhodamine B dye to 2 g of hydrogel and then stirred until the color of the dye was uniform.

### 2.7 *In vitro* biocompatibility

Fibroblast L929 (from Nanchang University) was used to determine the cytotoxicity of hydrogel by means of the MTT method (Wang et al., 2016; Guo et al., 2019). The lyophilized material was cultured in DMEM containing 10% fetal bovine serum for 24 h to obtain an extract (60 mg/mL). L929 cells were seeded in 96-well plates at a concentration of 4,000 cells per well and incubated for 24 h. Then, the medium was removed and replaced with 100 μL of leaching solution. After culturing for 1 day, 3 days, and 7 days, the medium was replaced with 90 μL of medium and 10 μL of CCK-8 reagent and incubated in an incubator for 2 h. Finally, the 96-well plate was removed. Absorbance values were measured at 450 nm by the microplate reader (Bio-Rad, United States).

In live/dead staining experiments (Qu et al., 2017; Lu et al., 2022), cells were inoculated in 24-well plates and incubated for 24 h at a concentration of 10,000 cells per well. Then, the medium was removed and changed to 100 μL of leaching solution. After 7 days of incubation, we prepared the live/dead staining solution in the dark; 5 µL of PI and 15 µL of AM were added to 5 mL of PBS, shaken, and mixed. We aspirated the medium in the 24-well plate, added 200 µL of the working solution to each plate, incubated them in the dark for 30 min, and then observed the solution with a fluorescence microscope. Under dark conditions, the cells were stained under a fluorescence microscope at the wavelengths of 470 nm (green for living cells) and 525 nm (red for dead cells).

### 2.8 Testing of antibacterial properties of materials

Antibacterial activity was measured by means of the coating method, and surface antibacterial activity was tested using *Escherichia coli* (*E. coli*) and *Staphylococcus aureus* (*S. aureus*) (Tian et al., 2018). We weighed out 20 mg of UV-sterilized freeze-dried 7/3 CPH, 5/5 CPH, 3/7 CPH hydrogels, CMCS, and PEI materials, placed them in 5 mL centrifuge tubes, and added 3 mL of the bacterial culture medium to prepare extracts (6.67 mg/mL); 50 µL of *Escherichia coli* was added to the extract, and the group without adding materials was the blank control group. After sealing with parafilm, the culture was incubated on a shaker at 37°C at 100 rpm for 6 h and then diluted to 10^–5^, and plated in three parallel plates for each sample after sealing with parafilm. After 18 h at 37°C, colony forming units (CFUs) were counted on the Petri dish. The antibacterial performance test procedure of *Staphylococcus aureus* is the same as that of *Escherichia coli*.

In bacterial shooting electron microscopy (Xie et al., 2020), after incubating the material with bacteria for a certain time, we aspirated 1 mL of bacterial solution and centrifuged it at 8,000 rpm for 1 min, and then added 1 mL of PBS to wash it twice. Then, we discarded the supernatant and added 2.5% glutaraldehyde to mix it well and kept it overnight at 4°C. After washing with PBS, we added 30%, 50%, 70%, 80%, 90%, and 100% ethanol solution successively to perform gradient dehydration and drying. The morphology of bacteria was observed using a scanning electron microscope, and the group without adding materials was the blank control group.

### 2.9 *In vivo* animal wound healing experiments

Female SD rats (7–8 weeks old) were anesthetized by injection of 3 wt% sodium pentobarbital and fixed on surgical cork boards. The rats were divided into two groups at random: the blank control group (0.2 mL of normal saline) and the hydrogel treatment group (0.2 mL each of 7/3 CPH, 5/5 CPH, and 3/7 CPH). After hair removal, a full-thickness skin lesion with a diameter of 10 mm was created on the back of the rat, and 0.2 mL of each group of materials was added. Water and food were given regularly, the size of the wound site was recorded on days 3, 7, and 14, and the closure rate was counted by the following formula:

$$closure\;rate\left(\%\right)=\frac{S_{0-}S_{t}}{S_{0}}x100\left(\%\right)$$

Among them, S_0_ represents the original size of the wound, and S_t_ represents the wound area on the day of recording.

### 2.10 Observation of material–tissue interface by electron microscopy

The tissue samples of the 5/5 CPH material group were taken on the third day and kept overnight at 4°C after adding 2.5% glutaraldehyde under gradient dehydration with ethanol and freeze-drying. The morphology of the interface between the material and the tissue was observed through a scanning electron microscope.

### 2.11 Histopathological study of wounds

On the 14th day, the wound tissues of each group were taken and immersed in 4% paraformaldehyde tissue fixative solution for 24 h and then embedded in paraffin and stained with hematoxylin–eosin (HE) and Masson’s trichrome to prepare 5 μm-thick tissue slices. Slices were imaged and quantitatively analyzed with a light microscope. Histological skin samples were evaluated for collagen density and the number of new blood vessels through ImageJ software. In terms of animal experiments, at least three biological replicates were performed in each group, and HE and Masson staining was conducted, respectively.

### 2.12 Histopathological study of wounds

For all the experiments, at least three samples were examined, and the experiments were repeated at least three times. All results were presented as means ± standard deviation (*p < 0.05, **p < 0.01, and ****p < 0.0001). One-way or two-way analysis of variance (ANOVA) was performed for data analysis in GraphPad Prism 8.0 or Origin.

## 3 Results and discussion

### 3.1 Preparation and characterization of CPH

**FIGURE 1:**
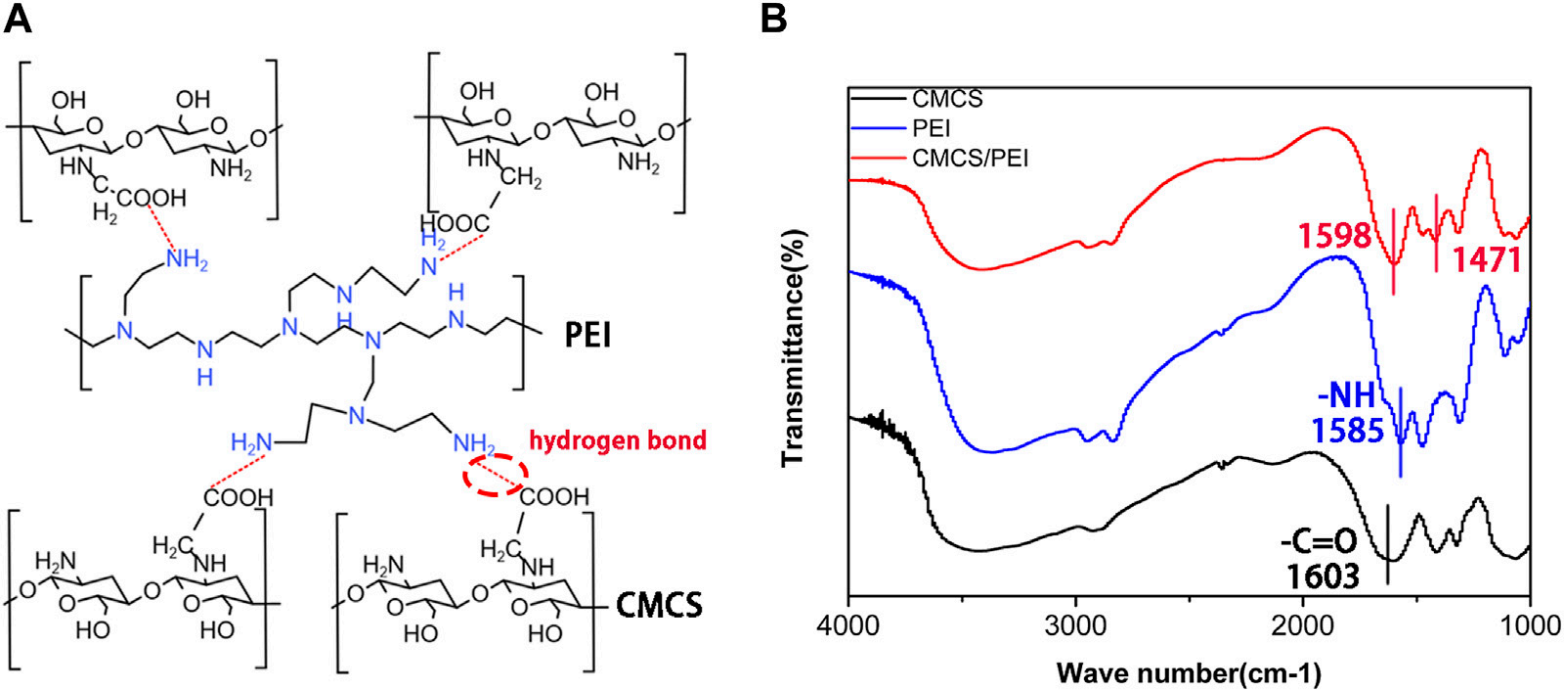
**(A)** Schematic diagram of the cross-linking mechanism of the hydrogel and **(B)** Fourier infrared of CMCS, PEI, and CMCS/PEI hydrogels.

Briefly, CPH hydrogels combined with aqueous CMCS (degree of substitution: ≥90%; isoelectric point: 3–4; 5 wt%) and PEI (Mw, ca. 70,000; 5 wt%) solutions were thoroughly mixed and stirred at different volumes (7:3; 5:5; and 3:7). The mixture was freeze-dried and then ground into powder for storage, and the hydrogel was formed after adding water and standing. The schematic diagram of the cross-linking structure of CPH hydrogels is shown in Figure 1A, and the gelation method of CMCS and PEI is hydrogen-bond cross-linking. In CMCS, PEI, and CPH FTIR spectroscopy analysis, the peak of carboxylic acid (─COOH) in CMCS was found to be located at 1,603 cm^−1^, and the peak of amine group (─NH_2_ or ─NH) in PEI was located at 1,585 cm^−1^ and moved to 1598 cm^−1^ and 1,471 cm^−1^ in the CPH gel, respectively. The shift in the characteristic carboxylic acid and amine peaks in the CPH gel did not generate new characteristic peaks, suggesting that CMCS and PEI are cross-linked through physical interactions (such as hydrogen bonding and electrostatic interactions) rather than covalent bonds (Figure 1B) (Peng et al., 2021).

### 3.2 Characterization of CPH

**FIGURE 2:**
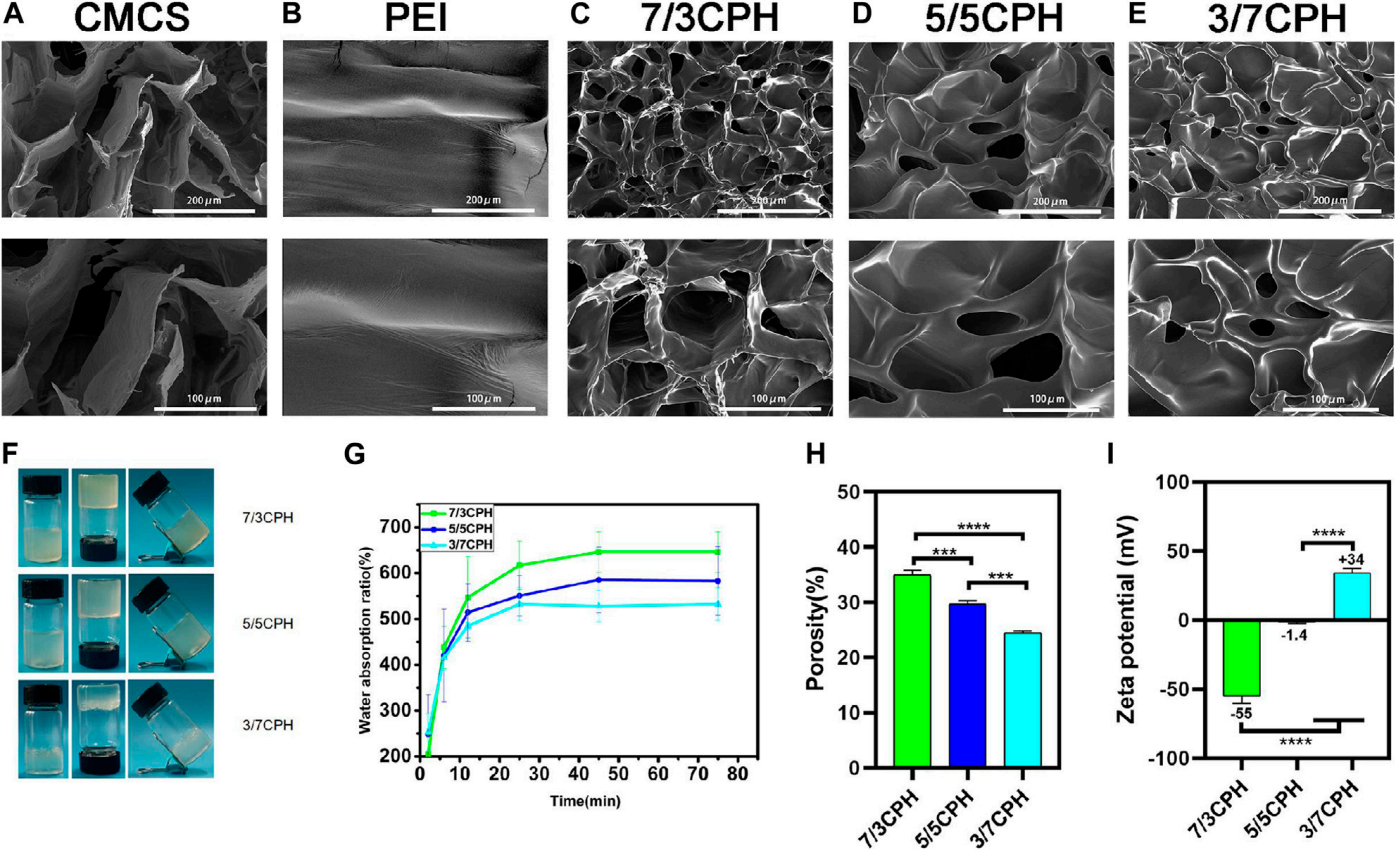
**(A–E)** Electron microscope images of CMCS, PEI, 7/3 CPH, 5/5 CPH, and 3/7 CPH hydrogel materials. **(F)** General image of the gel. **(G)** Water absorption of the hydrogel. **(H)** Hydrogel porosity. **(I)** Hydrogel zeta potential. *n* = 3; significance levels were set at **: *p* < 0.01, ***: *p* < 0.001, and ****: *p* < 0.0001.

The morphology of the hydrogel was observed under an electron microscope, and it can be seen that the CMCS and CPH hydrogel has a certain pore structure (Figures 2A–E). The pore structure of the material causes the material to have a certain degree of permeability, which is also conducive to wound healing. After gelation, the three different groups of hydrogels all showed a homogeneous transparent gel-like structure and maintained good stability for a long time (Figure 2F). By measuring the water absorption rate of the hydrogel, it can be seen that the water absorption rate of 7/3 CPH group was greater than that of the 5/5 CPH group, which in turn was greater than that of the 3/7 CPH group, that is, with the increase in CMCS content, the water absorption rate increases (Figure 2G). The hydrogel’s porosity was measured by the liquid displacement method, and it can be seen that the material’s porosity decreases with the decrease in CMCS content (Figure 2H). Through the zeta potential analysis of the three materials, the potential of 7/3 CPH is found to be around −55 mV, the potential of 5/5 CPH is around −1.4 mV, and the potential of 3/7 CPH is around +34 mV (Figure 2I), indicating that the potential increase of the hydrogel is related to the positively charged PEI. The 5/5 CPH group with the best zeta potential and the 7/3 CPH and 3/7 CPH groups have good stability (Peng et al., 2021; Yang et al., 2022).

### 3.3 Adhesion of hydrogel

**FIGURE 3:**
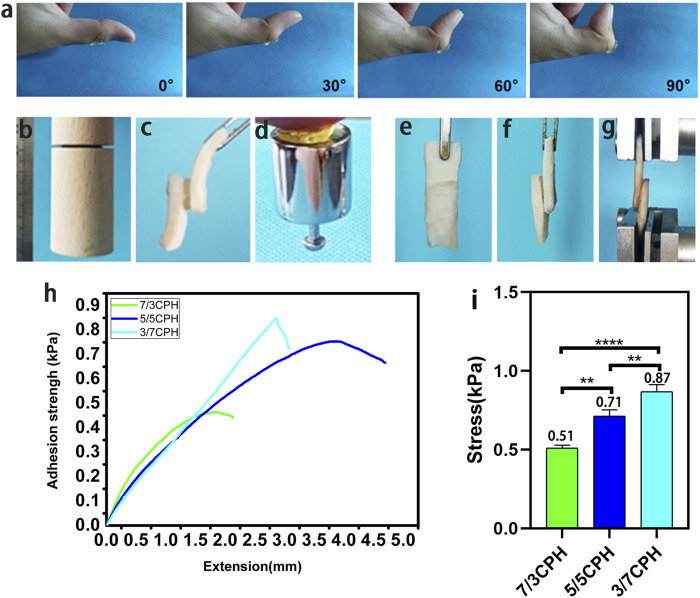
Adhesion of the hydrogel. **(A)** Stability of the material adhering to the thumb. **(B–D)** Image of the material adhering to different substrate surfaces (wood, pigskin, and weights). **(E–G)** Schematic diagram of shear test of material adhered to pigskin. **(H–I)** Shear test results. *n* = 3, significance levels were set at **: *p* < 0.01 and ****: *p* < 0.0001.

The general graph of the adhesion performance of the 5/5 CPH hydrogel shows that this hydrogel has good adhesion and adaptability in the process of bending the thumb joint from 0° to 90°. The hydrogel has certain toughness, which can withstand deformation caused by the movement of the skin on the surface of the organ and will not rupture to produce small hydrogel fragments without being subjected to great strain (Figure 3A). The material has good adhesion to different material surfaces (wood, pigskin, and weights) (Figures 3B–D). The adhesion was 0.51 kPa in the 7/3 CPH group, 0.71 kPa in the 5/5 CPH group, and 0.87 kPa in the 3/7 CPH(Figures 3H–I). The shear test showed (Figures 3E–I) that the 3/7 CPH group had the best adhesion and that the 7/3 CPH group had the worst adhesion, indicating that the adhesion of the material was mainly affected by the content of PEI. The material’s good adhesion performance may be due to the fact that the material is rich in active groups such as ─NH_2_, ─COOH, and ─OH, which can form electrostatic interactions and hydrogen bonds on the surface (Chen et al., 2021; Xie et al., 2022).

### 3.4 Rheological properties, injectability, and water shock stability of hydrogels

**FIGURE 4:**
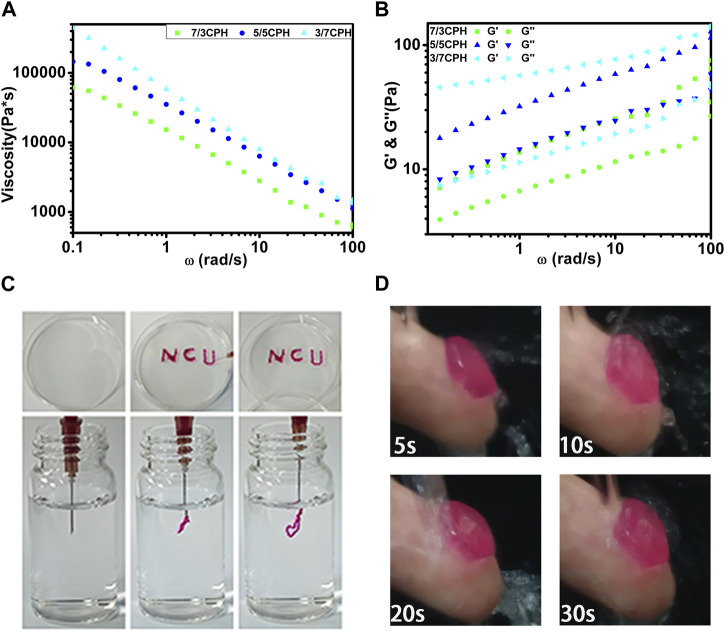
**(A)** Material shear viscosity as a function of *ω*. **(B)** Materials’ G^′^ and G^″^ as a function of *ω*. **(C)** The hydrogel was extruded through a needle for writing and injection using 5/5 CPH as ink. **(D)** A 5/5 CPH hydrogel disc was attached to the index finger and subjected to a stream of water for 30 s.

The degree of cross-linking of different proportions of materials is the main factor affecting the rheological properties of the materials. Oscillation frequency sweep measurements were used to evaluate the rheological properties of different groups of hydrogels. The viscosity of all groups of hydrogels decreased significantly with increasing *ω* and exhibited typical shear-thinning behavior, which reflects the typical characteristics of injectable hydrogels (Figure 4A) (Fu et al., 2022). The injectability is caused by the reversibility of non-covalent bonds such as hydrogen bonds, which are temporarily degraded by shear force during injection and easily reconstructed *in situ* at the injection site (Dimatteo et al., 2018; Lee, 2018). Hydrogels with injectable properties offer various advantages such as the ease of handling and minimal invasiveness when used as wound dressings. Figure 4C also intuitively shows the excellent injectable properties of the hydrogel. The hydrogel containing a small amount of rhodamine B can easily write the letters “NCU.” Underwater injection also shows that the hydrogel has excellent injectable properties (Figure 4C). As the frequency increased from 0.1 to 100 rad/s, G^′^ in each group was greater than G^″^, indicating that the material in each group has good stability (Figure 4B) (Guo et al., 2022). G^′^ of 3/7 CPH hydrogel was significantly increased compared with 7/3 CPH, indicating that 3/7 CPH hydrogel has better mechanical properties (Chen et al., 2021; Liu et al., 2022). A 5/5 CPH hydrogel disc with a diameter of 1 cm was attached to the index finger and impacted with water for 30 s to test the water impact stability of the material (Figure 4D; Supplementary Video S1), indicating that the material has good resistance to water impact (Chen et al., 2020).

### 3.5 *In vitro* biocompatibility of materials

**FIGURE 5:**
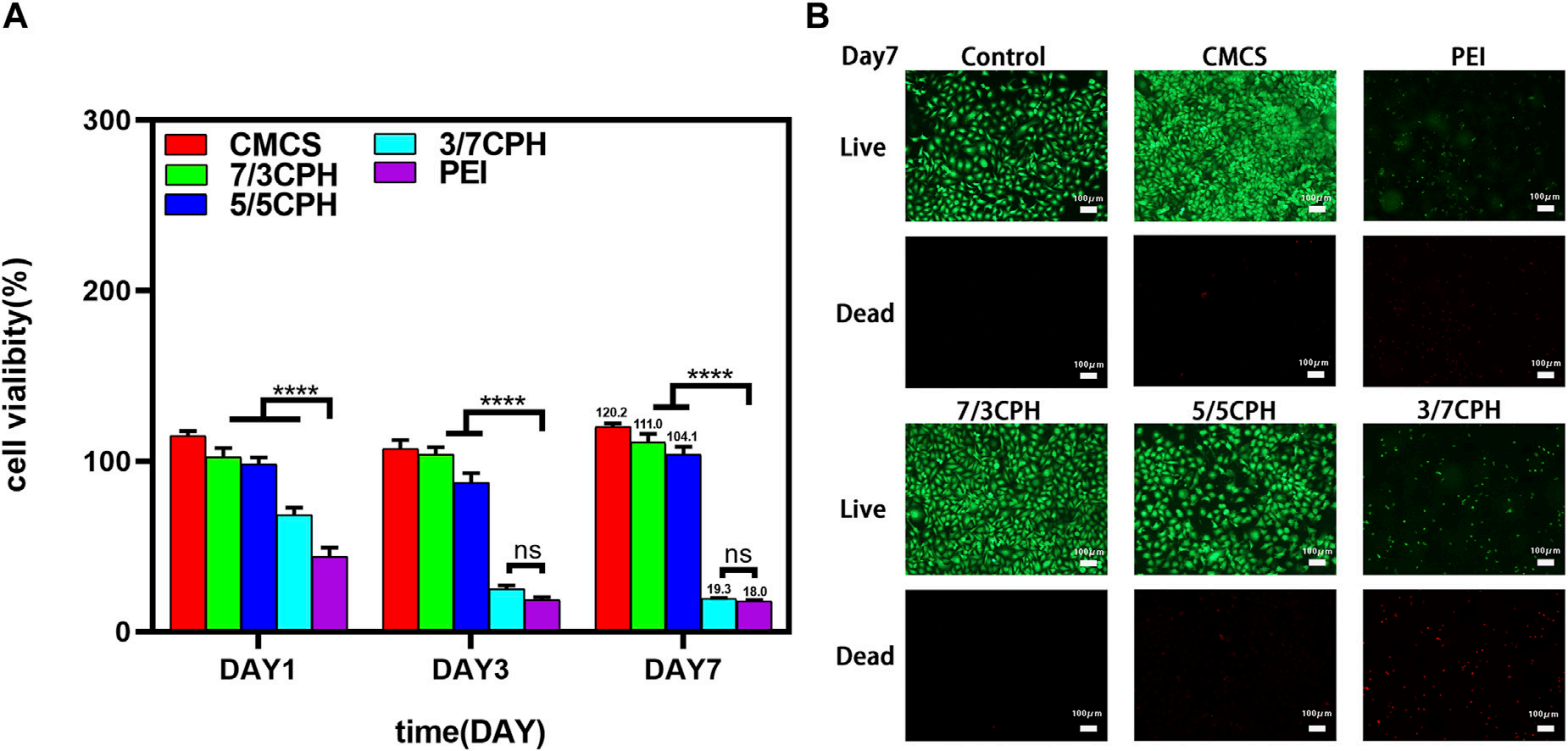
Biocompatibility of the hydrogel. **(A)** CCK-8 experiment of the material. **(B)** Day 7 live/dead staining experiment of the material. *n* = 3; significance levels were set at ns: *p* > 0.05 and ****: *p* < 0.0001.

The *in vitro* cytotoxicity of CPH hydrogels was first determined by the MTT method. In the MTT cell experiment, the survival rates were 111.0% in the 7/3 CPH group and 104.1% in the 5/5 CPH group on the seventh day *in vitro* (Figure 5A). It can be seen that CMCS, 7/3, and 5/5 have good biocompatibility, among which CMCS and 7/3 CPH hydrogels have obvious effects on promoting the growth of fibroblasts (as shown in Figure 5A). Cytotoxicity increased with the rise in PEI concentration, and the 3/7 CPH and pure PEI groups had greater cytotoxicity, which may be related to the toxicity of amino-rich PEI itself (Li et al., 2017; Liang et al., 2019; Peng et al., 2021). The results indicate that CMCS can reduce the cytotoxicity of PEI, and the 7/3 CPH and 5/5 CPH hydrogels have good biocompatibility. In live/dead staining on day 7, green represents live cells and red represents dead cells, showing that there are few dead cells in the control group, CMCS group, 7/3 CPH group, and 5/5 CPH group, while there are many red dead cells in the 3/7 CPH group and PEI group. The cell density of 7/3 CPH and 5/5 CPH is higher than that of the control group, which also indicates that 7/3 CPH and 5/5 CPH have a certain effect on promoting the growth of fibroblasts (Figure 5B). Therefore, this CPH hydrogel can serve as a potential candidate for biomaterial applications.

### 3.6 Evaluation of antibacterial properties of materials

**FIGURE 6:**
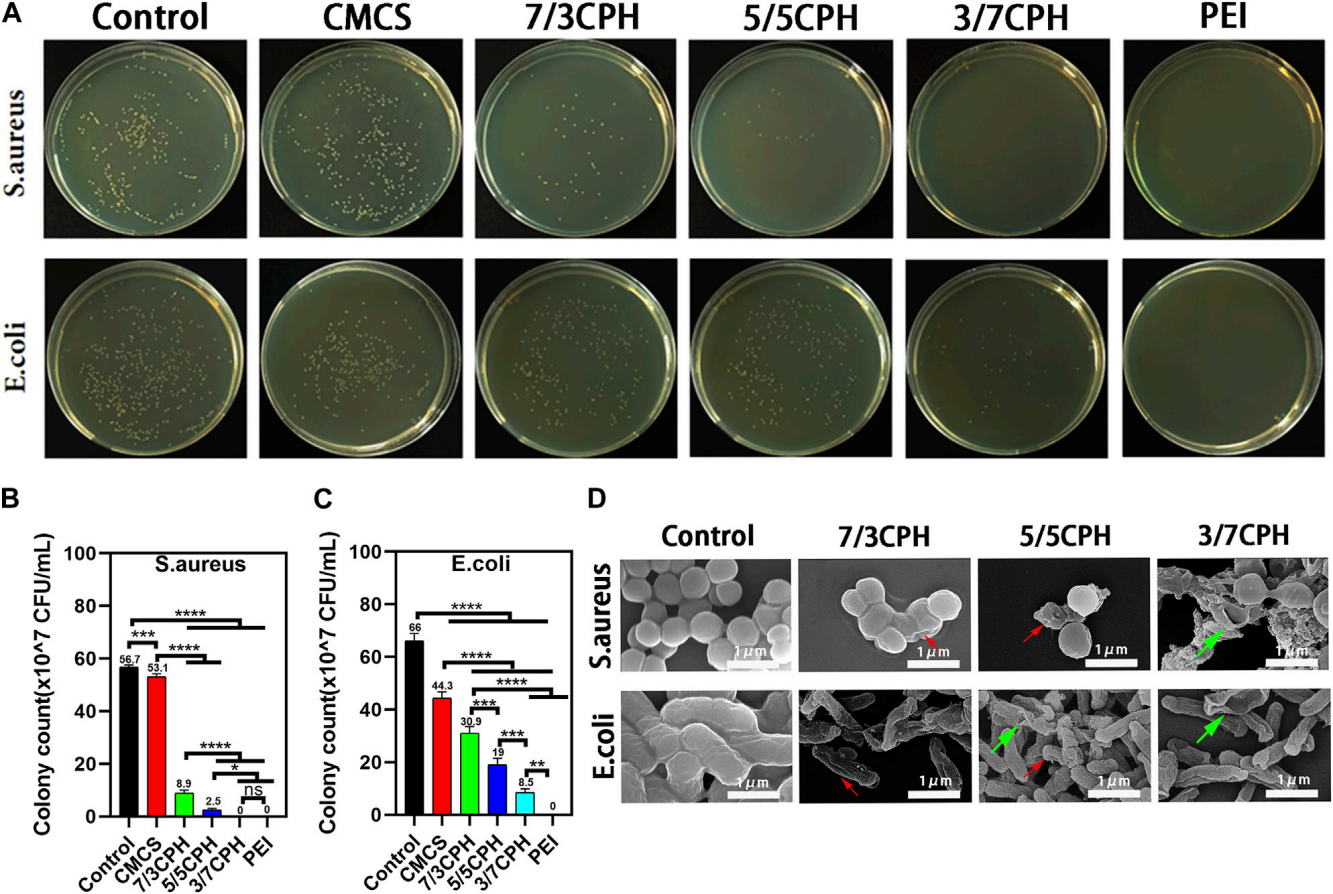
Antibacterial properties of materials. **(A)** Photograph of bacterial colonies on agar plates. **(B,C)** Summary of antibacterial ratios of *Staphylococcus aureus* and *Escherichia coli*. **(D)** Antibacterial electron microscope image of the material. *n* = 3; significance levels were set at ns: *p* > 0.05, *: *p* < 0.05, **: *p* < 0.01, ***: *p* < 0.001, and ****: *p* < 0.0001.

The antibacterial properties of CPH were both evaluated with *Staphylococcus aureus* (Gram-positive bacteria) and *Escherichia coli* (Gram-negative bacteria), and the corresponding CFUs were imaged to evaluate antibacterial ability. Compared to the blank control group, it can be seen that the CMCS group, 7/3 CPH, 5/5 CPH, and 3/7 CPH material group and PEI group have gradually stronger antibacterial ability against *Staphylococcus aureus* and *Escherichia coli*, and the strongest antibacterial is observed for the pure PEI group (both 100%). The CFUs of *Staphylococcus aureus* were 56.7 × 10^7^ CFU/mL in the blank group, 8.7 × 10^7^ CFU/mL in the 7/3 CPH group, 2.5 × 10^7^ CFU/mL in the 5/5 CPH group, and 0 CFU/mL in the 3/7 CPH group *in vitro*. The CFUs of *Escherichia coli* were 66.0 × 10^7^ CFU/mL in the blank group, 30.9 × 10^7^ CFU/mL in the 7/3 CPH group, 8.5 × 10^7^ CFU/mL in the 5/5 CPH group, and 19.0 × 10^7^ CFU/mL in the 3/7 CPH group (Figures 6B, C). This shows that, with the increase in PEI content, the antibacterial effect of the material is also more evident, and the antibacterial effect of PEI may be due to the cationic bactericidal effect of PEI rich in amino groups (Wang et al., 2016; Guo et al., 2019; Lu et al., 2022). In the material group, CMCS had weak antibacterial effects against both *Staphylococcus aureus* and *Escherichia coli* (both <50%) (Figures 6A–C), possibly because of the cationic antibacterial effect of a small number of amino groups that remained in CMCS (Qu et al., 2017). All three CPH materials had good antibacterial effects on *Staphylococcus aureus* and *Escherichia coli* (Figures 6B, C). Figure 6D shows the antibacterial electron microscope image of the material. The morphological changes of the bacteria after the interaction between the material and the bacteria for 0.5 h can be seen (red arrows represent wrinkles and green arrows represent rupture).

### 3.7 Evaluation of wound healing *in vivo*

**FIGURE 7:**
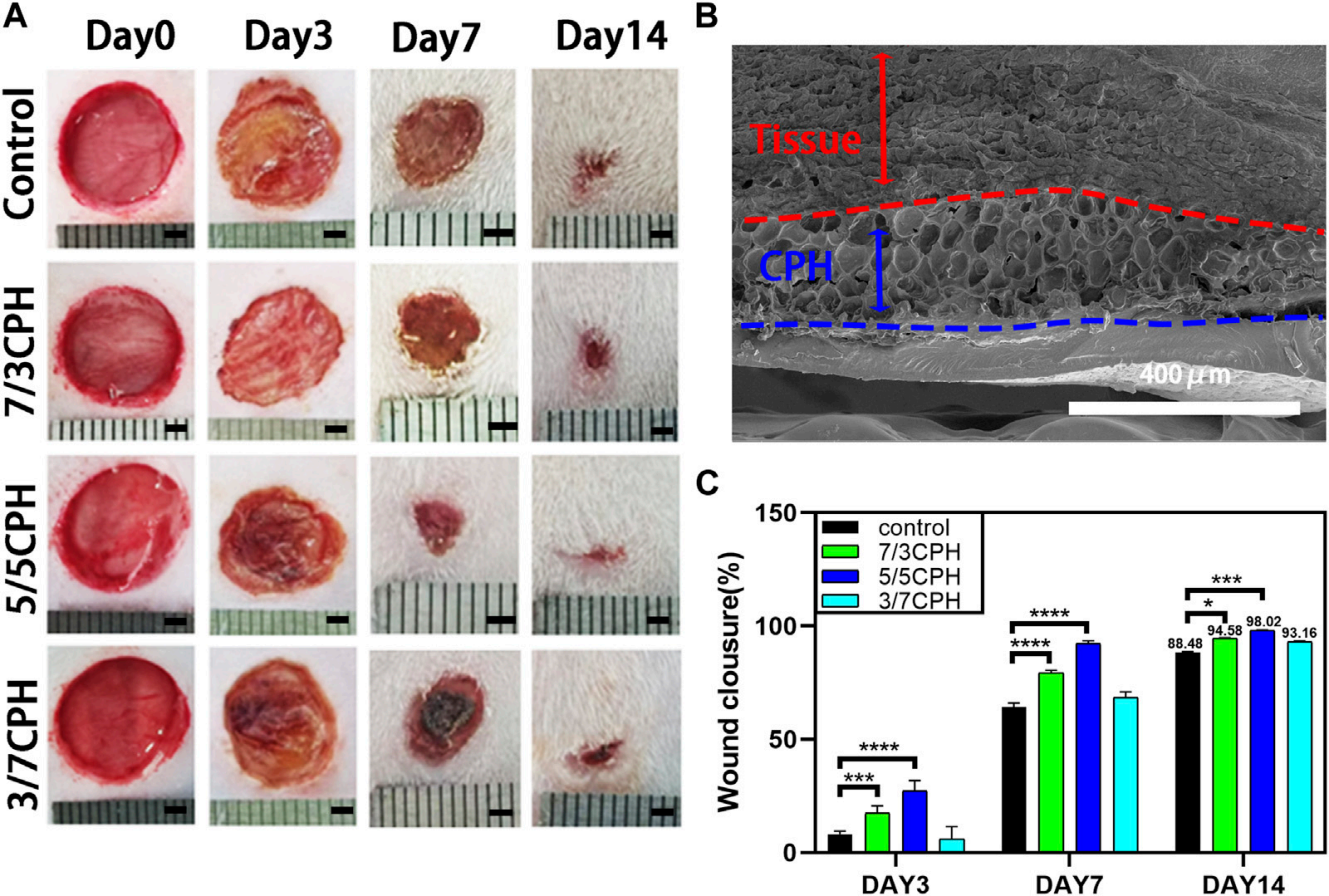
**(A)** Optical image of the wound healing process. Scale bar: 2 mm. **(B)** Electron micrograph of the tissue–material interface on day 3 of 5/5 CPH. **(C)** Comparison of wound healing on days 3, 7, and 14 for blank, 7/3 CPH, 5/5 CPH, and 3/7 CPH. *n* = 3; significance levels were set at *: *p* < 0.05, * **: *p* < 0.001, and ****: *p* < 0.0001.

The wound healing effect of the hydrogel was evaluated through the full-thickness skin defect model. SD rat dorsal skin wounds with a diameter of 10 mm were prepared with a skin punch, and 0.2 mL of materials in different proportions was uniformly injected onto the wounds with a syringe. The wounds in the 0.2 mL PBS group were used as blank controls (Figure 7A). On the third day, the wounds in each group showed a certain degree of shrinkage, and on the seventh day, the wound area in each group was reduced. The healing efficiency of the 7/3 CPH and 5/5 CPH groups was higher than that of the blank control, while the 5/5 CPH group was better than the 7/3 CPH group. After 14 days of healing, there were still scars in the wounds in each group. In the wound healing experiment, the healing rates were 88.48% in the blank control group, 94.58% in the 7/3 CPH group, 98.02% in the 5/5 CPH group, and 93.16% in the 3/7 CPH group (Figure 7C). The wounds treated with 7/3 CPH and 5/5 CPH hydrogels were better than those in the control group, showing the best therapeutic effect in the whole wound healing stage (Figure 7C), and the overall healing effect of the 5/5 CPH group was better than that of the 7/3 CPH group, which is inconsistent with the description of the MTT experiment. It is speculated that the actual healing environment in the body is complex. There are many factors such as bacteria and immunity in the rats raised under ordinary environment, meaning the final treatment effect of the 5/5 CPH group may be better than that of the 7/3 CPH group. In addition, Figure 5 shows that the 3/7 CPH group has significant cytotoxicity, while the 3/7 CPH group has significant wound closure (Figure 7C). On the one hand, it may be affected by multiple factors such as immunity and bacteria; on the other hand, it may also be that PEI has a good cell adhesion effect (Alba et al., 2022), leading to significant wound closure (Nagahama et al., 2021).Figure 7B shows the tissue–material interface electron microscope image of the material group on the third day. The dense structure is the tissue (the area indicated by the red arrow), and the loose porous structure is the hydrogel material (the area indicated by the blue arrow). It shows that the material has good adhesiveness to the tissue, and the material will not fall off during the wound healing process.

### 3.8 In terms of histological analysis

**FIGURE 8:**
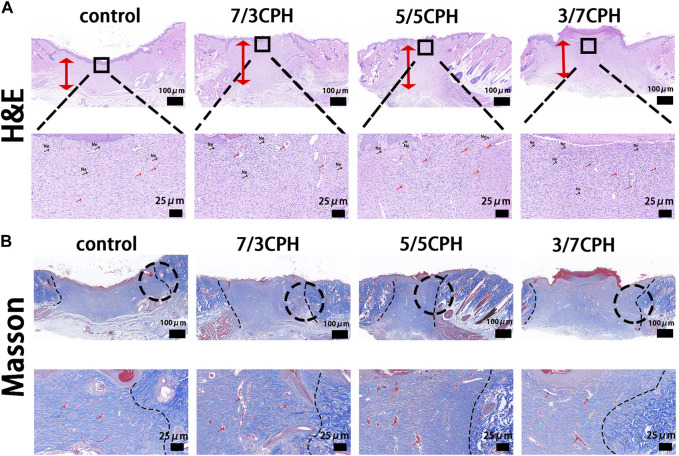
Histological analysis after 14 days.**(A)** hematoxylin–eosin (HE) and **(B)** Masson’s trichrome. Of note, the red bidirectional arrows indicate tissue healing thickness, while the blue and red arrows indicate collagen fibers and reconstructed blood vessels, respectively. Ne, neutrophils.

In order to further measure the wound healing effect of hydrogel, the wound tissue on day 14 was evaluated by HE and Masson staining. In HE staining, the thickness of the granulation tissue was first assessed (indicated by the red double-headed arrows). On day 14, 7/3 CPH and 5/5 CPH hydrogel-treated wounds significantly displayed the thickest granulation tissue, and a large number of new blood vessels were seen (indicated by the red arrows). In addition, 7/3 and 5/5 CPH hydrogels had less inflammatory cell infiltration on day 14 compared with the other two groups (indicated by black arrows) (Figure 8A). There were more hair follicles and accessory structures in those treated with 5/5 CPH hydrogels (Figure 8A). In Masson staining, as collagen fibers were stained blue, while muscle fibers and cytoplasm were stained red, more collagen precipitation was observed in the 7/3 CPH and 5/5 CPH groups compared to the control group and the 3/7 CPH group (blue arrow) (Figure 8B) (Zhou et al., 2017; Li et al., 2018; Cai et al., 2019).

## 4 Conclusion

In conclusion, in this work, we exploited the strong physical interaction between CMCS and PEI to form novel CMCS/PEI (CPH) hydrogels with different ratios, mainly to explore the injectability, healing promotion, antibacterial properties, adhesion, and rheology of the material and the wound healing ability *in vivo*. At the wound surface, it was found that 7/3 CPH and 5/5 CPH hydrogels can promote wound healing well and have good antibacterial properties, and they are injectable and have certain adhesion as dressings. In this study, with a wide range of natural sources, the cost of chitosan is low. The preparation method of the hydrogels is simple but time-consuming. Hydrogel wound dressings have good application prospects.

## Data Availability

The raw data supporting the conclusion of this article will be made available by the authors, without undue reservation.
